# Biopsychosocial Correlates of Cognitive Function Among Korean Older Adults: History of Hypertension and Diabetes

**DOI:** 10.1093/geroni/igab046.3469

**Published:** 2021-12-17

**Authors:** Kyuyoung Cho, Hye Won Chai

**Affiliations:** 1 Dong-A University, Busan, Pusan-jikhalsi, Republic of Korea; 2 The University of Texas at Austin, Austin, Texas, United States

## Abstract

Based on biopsychosocial perspectives on health, this study examined risk and protective factors of cognitive function among Korean older adults. Specifically, we focused on comparing the role of these factors based on the respondents' history of having hypertension or diabetes. This study used 2009 Korean National Health Insurance Service data that included a sample of older adults who maintained qualification for health insurance and medical aid in 2002 (n=26,242). Cognitive function was measured using KDSQ-C and biopsychosocial factors included metabolic syndrome, drinking, smoking, and walking. The sample was divided into two groups based on their medical history, and thus four sets of linear regression models were analyzed to explore the associations between biopsychosocial factors and cognitive functioning. Among individuals with a history of hypertension, metabolic syndrome, drinking, and walking were associated with cognitive functioning. For those without a history of hypertension, only drinking and walking were associated with cognitive functioning. For diabetes, smoking and walking were associated with cognitive functioning among older adults with a history of diabetes. For those without a history of diabetes, drinking and walking were associated with cognitive functioning. In sum, metabolic syndrome was a particularly significant correlate of cognitive function among Korean older adults with a history of hypertension. Walking was a consistently significant factor regardless of medical history. These results highlight the importance of considering medical history of chronic conditions such as hypertension and diabetes in identifying factors associated with older adults' cognitive function and further developing tailored prevention programs for cognitive decline.

